# Supplementary material for: Small RNA Profiling of piRNAs in Colorectal Cancer Identifies Consistent Overexpression of piR-24000 That Correlates Clinically with an Aggressive Disease Phenotype
Source: Cancers (Basel). 2020 Jan 12;12(1):188. doi: 10.3390/cancers12010188 (PMC7016796; doi:10.3390/cancers12010188)
Supplement: Supplementary file 1 [file cancers-12-00188-s001.pdf]

Table S1a: Significantly expressed upregulated (fold change > 2) piRNAs in CRC (N=32)		
**Feature ID**	**Fold change (TUMOR vs. NORMAL)**	***p-value* (TUMOR vs. NORMAL)**
piR-hsa-2107	23.12	2.10E-07
piR-hsa-28160	15.36	1.18E-04
piR-hsa-27138	13.58	5.99E-03
piR-hsa-24684	11.58	1.72E-04
piR-hsa-27621	6.77	1.51E-02
piR-hsa-1243	4.72	2.96E-02
piR-hsa-7116	4.65	8.60E-05
piR-hsa-1282	4.32	1.96E-03
piR-hsa-26589	3.98	3.32E-03
piR-hsa-26591	3.98	3.32E-03
piR-hsa-7193	3.96	5.70E-10
piR-hsa-29715	3.64	1.78E-03
piR-hsa-24672	3.62	3.28E-09
piR-hsa-5937	3.60	4.08E-09
piR-hsa-5938	3.57	3.92E-09
piR-hsa-18860	3.22	1.48E-02
piR-hsa-5934	3.19	4.25E-04
piR-hsa-9491	3.16	1.72E-03
piR-hsa-24000	3.16	9.44E-03
piR-hsa-5936	3.14	1.58E-04
piR-hsa-27622	2.91	4.83E-07
piR-hsa-27623	2.91	4.83E-07
piR-hsa-27007	2.84	1.19E-03
piR-hsa-23230	2.63	4.66E-04
piR-hsa-23231	2.63	4.66E-04
piR-hsa-28190	2.60	1.23E-05
piR-hsa-28299	2.38	3.18E-02
piR-hsa-6147	2.33	3.42E-02
piR-hsa-1593	2.29	3.91E-02
piR-hsa-32170	2.16	1.57E-02
piR-hsa-28590	2.08	3.44E-02
piR-hsa-21126	2.05	2.55E-02

Formatted: Font: Italic

Table S1b: Significantly expressed downregulated (fold change ≤ 2) piRNAs in CRC (N=111)

Feature ID	Fold change (TUMOR vs. NORMAL)	***pP-value*** **(TUMOR vs. NORMAL)**
piR-hsa-25779	-95.02	2.53E-02
piR-hsa-24683	-87.53	2.32E-04
piR-hsa-32204	-73.74	5.42E-05
piR-hsa-32184	-41.57	1.20E-03
piR-hsa-2106	-40.78	8.41E-05
piR-hsa-16034	-32.18	1.38E-03
piR-hsa-12682	-28.13	1.67E-02
piR-hsa-14219	-26.74	5.05E-03
piR-hsa-32458	-25.50	2.66E-03
piR-hsa-32236	-25.00	7.72E-03
piR-hsa-26946	-22.08	3.02E-04
piR-hsa-27619	-21.15	2.75E-03
piR-hsa-5770	-21.15	2.75E-03
piR-hsa-2154	-20.93	2.76E-05
piR-hsa-32161	-20.82	9.90E-03
piR-hsa-32162	-17.34	6.74E-03
piR-hsa-20757	-14.35	5.87E-03
piR-hsa-28845	-13.59	9.33E-08
piR-hsa-32187	-13.44	2.41E-02
piR-hsa-21238	-12.90	2.37E-03
piR-hsa-1281	-12.79	2.30E-02
piR-hsa-442	-12.28	1.86E-02
piR-hsa-6145	-11.10	1.46E-02
piR-hsa-15449	-11.05	3.29E-02
piR-hsa-26508	-10.76	2.51E-02
piR-hsa-1580	-10.65	2.09E-03
piR-hsa-18677	-10.58	1.96E-03
piR-hsa-31788	-10.51	1.88E-02
piR-hsa-28019	-10.46	5.87E-03
piR-hsa-17520	-8.86	1.67E-02
piR-hsa-18905	-8.27	4.07E-03
piR-hsa-16035	-8.05	4.55E-04
piR-hsa-11362	-7.57	3.59E-07
piR-hsa-27134	-7.43	3.26E-02
piR-hsa-26399	-7.00	1.80E-02
piR-hsa-23621	-6.78	1.25E-02
piR-hsa-4276	-6.61	1.66E-02
piR-hsa-1861	-6.44	2.43E-02
piR-hsa-13643	-6.33	2.57E-03

Formatted: Font: Italic

piR-hsa-14647	-6.15	1.11E-10
piR-hsa-27730	-5.89	6.94E-07
piR-hsa-22381	-5.60	6.71E-04
piR-hsa-27729	-5.37	7.99E-06
piR-hsa-25783	-5.36	2.46E-02
piR-hsa-12525	-5.12	3.39E-02
piR-hsa-1710	-5.04	1.56E-08
piR-hsa-28117	-5.02	4.39E-03
piR-hsa-17946	-4.87	1.79E-02
piR-hsa-7646	-4.86	7.48E-03
piR-hsa-11361	-4.77	1.01E-09
piR-hsa-26803	-4.75	4.11E-12
piR-hsa-11360	-4.71	9.93E-10
piR-hsa-1251	-4.63	3.04E-02
piR-hsa-3645	-4.55	3.84E-02
piR-hsa-30636	-4.49	3.00E-02
piR-hsa-28186	-4.48	9.69E-03
piR-hsa-15022	-4.45	5.05E-03
piR-hsa-32299	-4.36	8.43E-04
piR-hsa-8030	-4.06	2.99E-03
piR-hsa-23617	-4.00	1.52E-05
piR-hsa-17560	-3.98	1.55E-06
piR-hsa-27615	-3.86	3.16E-03
piR-hsa-30734	-3.86	2.11E-02
piR-hsa-1043	-3.84	2.84E-04
piR-hsa-22382	-3.72	1.71E-02
piR-hsa-12759	-3.59	1.70E-03
piR-hsa-32492	-3.58	2.81E-02
piR-hsa-3200	-3.56	1.20E-07
piR-hsa-1361	-3.54	8.29E-07
piR-hsa-28521	-3.46	2.39E-02
piR-hsa-1944	-3.41	1.30E-07
piR-hsa-28472	-3.26	4.67E-02
piR-hsa-31237	-3.25	1.00E-02
piR-hsa-317	-3.23	7.94E-06
piR-hsa-28319	-3.20	1.01E-03
piR-hsa-32158	-3.20	3.16E-02
piR-hsa-28403	-3.12	4.81E-02
piR-hsa-28406	-3.12	4.81E-02
piR-hsa-23884	-3.12	4.70E-06
piR-hsa-9361	-3.07	2.12E-02
piR-hsa-820	-3.06	4.42E-03
piR-hsa-28846	-3.02	9.13E-05
piR-hsa-16404	-2.98	8.28E-03

piR-hsa-26570	-2.95	1.65E-02
piR-hsa-28646	-2.93	8.35E-03
piR-hsa-3178	-2.92	8.43E-04
piR-hsa-515	-2.89	4.04E-05
piR-hsa-27283	-2.88	6.75E-07
piR-hsa-1847	-2.82	2.42E-02
piR-hsa-5067	-2.75	3.91E-02
piR-hsa-27282	-2.70	9.01E-04
piR-hsa-12454	-2.68	2.54E-02
piR-hsa-21707	-2.54	4.22E-02
piR-hsa-952	-2.53	6.26E-04
piR-hsa-28212	-2.53	8.90E-05
piR-hsa-26524	-2.51	3.42E-03
piR-hsa-26525	-2.51	3.42E-03
piR-hsa-27616	-2.50	2.21E-03
piR-hsa-1742	-2.48	8.85E-04
piR-hsa-619	-2.48	7.11E-03
piR-hsa-26872	-2.44	2.91E-02
piR-hsa-27208	-2.43	3.24E-02
piR-hsa-32585	-2.39	9.74E-03
piR-hsa-29218	-2.37	4.25E-04
piR-hsa-32165	-2.32	3.49E-02
piR-hsa-2138	-2.27	1.71E-02
piR-hsa-12789	-2.26	4.19E-05
piR-hsa-26039	-2.24	3.42E-03
piR-hsa-32159	-2.19	2.90E-03
piR-hsa-28851	-2.15	4.90E-02
piR-hsa-25046	-2.07	2.29E-03

Table S2: Primer sequences used for reverse transcription (RT) and quantitative polymerase chain reaction (QPCR)	
**Gene name**	**Primer sequence (5’-3’)**
Oligo dT for reverse transcription	CAGGTCCAGTTTTTTTTTTTTTTTTVN
RNU6B Forward	CGCTTCGGCAGCACATATACTA
RNU6B Reverse	ACGCTTCACGAATTTGCGT
piR-hsa-24000 Forward	GCAGCTGCATCCACTGATAGAC
piR-hsa-24000 Reverse	GGTCCAGTTTTTTTTTTTTTTTATTGTTCAAG
